# Characterisation of Some Phytochemicals Extracted from Black Elder (*Sambucus nigra* L.) Flowers Subjected to Ozone Treatment

**DOI:** 10.3390/molecules26185548

**Published:** 2021-09-13

**Authors:** Natalia Matłok, Ireneusz Kapusta, Tomasz Piechowiak, Miłosz Zardzewiały, Józef Gorzelany, Maciej Balawejder

**Affiliations:** 1 Department of Food and Agriculture Production Engineering, University of Rzeszow, St. Zelwerowicza 4, 35-601 Rzeszów, Poland; milosz.zardzewialy@gmail.com (M.Z.); gorzelan@ur.edu.pl (J.G.); 2 Department of Food Technology and Human Nutrition, Rzeszów University, St. Zelwerowicza 4, 35-601 Rzeszów, Poland; ikapusta@ur.edu.pl; 3 Department of Chemistry and Food Toxicology, Collegium of Natural Sciences, University of Rzeszow, St. Ćwiklińskiej 1a, 35-601 Rzeszów, Poland; tomek.piechowiak92@gmail.com (T.P.); maciejb@ur.edu.pl (M.B.)

**Keywords:** *Sambuci flos*, ozonation, syrup, antioxidant activities, polyphenols, UPLC–PDA–MS/MS, quercetin 3-*O*-rutinoside, 3,4-dicaffeoylquinic acid

## Abstract

Elderflowers are a well-known source of bioactive compounds. The amount of isolated bioactive compounds may be increased by applying various abiotic and biotic factors. Gaseous ozone (10 and 100 ppm) was used in the process of preparing flowers. Next, the flowers were treated with sugar syrup to extract bioactive compounds. It was shown that this treatment, including the influence of extraction temperature, significantly affects the contents of polyphenols (liquid chromatography–mass spectrometry (LC–MS) methods) and vitamin C, as well as the antioxidant potential (cupric reducing antioxidant capacity (CUPRAC method)), the profile of volatile substances (head space–solid-phase microextraction (HS–SPME methods)) and the colour of the syrup (Commission Internationale de l’Eclairage (CIE) L*a*b* methods). The findings show that an increased dose of ozone and higher extraction temperature applied in the process of syrup production resulted in higher contents and different compositions of bioactive compounds. The highest contents of bioactive compounds were identified in syrup obtained from raw material treated with ozone for 15 min (concentration = 10 ppm) and extraction with sugar syrup at a temperature of 60 °C.

## 1. Introduction

Black elder (*Sambucus nigra* L.) is a deciduous shrub, the parts of which, i.e., leaves, berries, flowerheads, roots and shoots, as well as bark, are used for culinary and medicinal purposes [1]. The shrubs produce small white flowers arranged into umbels. Berries, dark purple to black in colour, develop from these in late summer and early autumn [2].

It has been established by researchers that *Sambucus nigra* L. is a natural source of beneficial biologically active elements that are used in traditional medicine [3,4,5]. Flowers of black elder containing flavonoids [6,7], polyphenols, organic acids and vitamins are helpful in preventing and treating numerous diseases [8,9]. Substances identified in flowers of black elder include kaempferol, quercetin, isoquercetin, rutoside [10] astragalin, rutin, hyperoside and nicotiflorin [11,12]. Furthermore, the flowers also contain phenolic acids and their glucosides, including caffeic, ferulic, chlorogenic and *p*-coumaric acids, as well as triterpenes [13], sterols [14] and essential oils (0.03–0.14%), with a high concentration of free fatty acids amounting to 65% [15]. Other substances identified in flowers of black elder include ascorbic, fumaric, citric, tartaric, valeric and malic acids [3].

Products obtained from black elder may be harmful to people [16] because of sambunigrin, which is a cyanogenic glycoside, present in the fruit and flowers [10,17,18]. It was shown, however, that thermal processing leads to a decrease in the contents of cyanogenic glycosides, including sambunigrin, which improves the safety of products intended for consumption. Products made from this raw material should be subjected to analysis, which will confirm the absence of cyanogenic glycosides [16,19].

The flavour of elderflowers is associated with volatile substances, and approximately 100 compounds of this kind were identified [20]. These compounds represent various groups, including hydrocarbons (terpenes) and ethers, as well as oxides, alcohols, acids and ketones [21].

Flowers of black elder are mainly processed into extracts [22], whereas elderberries are used in the food industry for the production of juice, jams, yoghurt and wine [2,23]. Because of the high contents of anthocyanins, elderberry juice is used in the food industry as a colour additive [24,25].

Controlled use of various biotic and abiotic factors (so-called stress factors) elicits a physiological response in the plant, which leads to the synthesis of increased amounts of some metabolites [26], which are frequently produced at a time of stress, as part of a defence system, e.g., antioxidants. Such factors include gaseous ozone, which has been shown to modify the quality of raw plant materials [27,28]. By applying gaseous ozone to plant material, it is possible to activate a number of metabolic pathways [29], which induce changes in the composition of bioactive compounds [30].

The study aimed to investigate the effects of ozone treatment applied to flowers of black elder on the composition of bioactive compounds, profile of volatile substances and colour of the sugar syrups produced from the elderflowers’ extraction. It has been hypothesised that ozone gas induces an increase in the content of bioactive compounds in the ozonated raw material, which will ultimately affect the quality of the sugar syrup produced from them.

## 2. Results and Discussion

### 2.1. Antioxidant Activity of the Syrups

Flowers of black elder used as plant material were subjected to ozone treatment. It was hypothesised that, by applying ozone treatment, it is possible to affect the contents of selected bioactive compounds in the material, in particular polyphenols and vitamin C, as well as antioxidant potential [27,31]. Components pass into syrup through extraction, and this way, they affect the profile of bioactive compounds.

**Figure 1:**
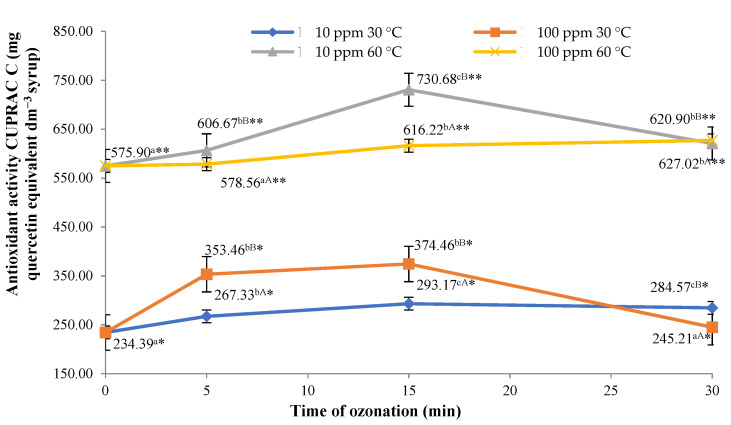
Antioxidant activity (CUPRAC method) identified in syrups from flowers of black elder, relative to the dose of ozone applied and temperature of sugar syrup used. Note: Differences in results between the time of ozonation (for certain ozone concentration) are indicated by different small letters, differences between ozone concentration (for certain ozonation time) are indicated by different capital letters and differences between sugar syrup temperatures are indicated by * or ** (for the same ozone-treatment conditions) at α < 0.05.

As shown in Figure 1, the effects of the procedure (ozonation of the material) confirmed the above hypothesis and included a change in the antioxidant potential (CUPRAC method) of the final syrups. All the syrups made from the material (flowerheads of black elder) treated with ozone were found to have higher antioxidant potential, compared to the syrup made from the control sample (flowerheads not treated with ozone). The highest antioxidant activity in the group of syrups produced with the use of sugar syrup with a temperature of 30 °C was identified in the product obtained from raw material treated with ozone for 15 min at a rate of 100 ppm. This syrup was found with an antioxidant potential of 374.5 mg quercetin equivalent in dm^3^ of the syrup, which was 59.8% higher than the antioxidant potential of the control syrup. Notably, by producing isolate from sugar syrup with a temperature of 30 °C or higher, it is possible to reduce cyanogenic glycoside—sambunigrin (benzaldehyde cyanohydrin glycoside), which is harmful to people and occurs in the raw material. It should be noted that cyanogenic glycoside is not always present in elderberry flowers [32]. Therefore, it can be presumed that these compounds were not present in the raw material analysed, which was confirmed by UPLC–MS analysis. The use of sugar syrup at a temperature of 60 °C resulted in the extraction of larger amounts of compounds, including antioxidants. Increasing the temperature of the extraction process usually results in an increased amount of extracted components [33].

The highest increase was also observed in the syrup produced from flower heads of black elder treated with ozone for 15 min at a rate of 10 ppm. A correlation was observed between the duration of the ozone treatment applied to the raw material and the antioxidant potential of the syrup produced. The observed potentials of the syrups investigated are also differentiated relative to the concentration of ozone applied in the treatment; however, these relationships were weaker than in the case of ozone treatment duration. Application of ozone in the initial processing of flowers of black elder used in the production of the syrups possibly led to onset of oxidative stress. This type of stress induces changes in the activity of antioxidant enzymes, in particular superoxide dismutase, ascorbate peroxidase and phenylalanine ammonialyase [34]. An increase in the antioxidant potential of marjoram following treatment with ozone at a rate of 1 ppm was reported by Matłok et al. [35]. Similar effects of the ozonation process were also observed in the case of blueberries [36] and raspberries [34].

### 2.2. Content of Vitamin C in Syrups

**Figure 2:**
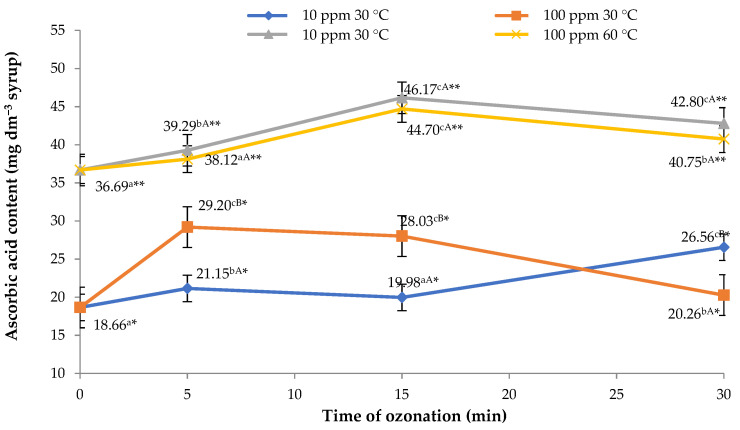
Contents of vitamin C in syrups from flowers of black elder, relative to the dose of ozone applied and temperature of sugar syrup used. Note: Differences in results between the time of ozonation (for certain ozone concentration) are indicated by different small letters, differences between ozone concentration (for certain ozonation time) are indicated by different capital letters and differences between sugar syrup temperatures are indicated by * or ** (for the same ozone-treatment conditions) at α < 0.05.

The most important bioactive components of the products obtained from flowerheads of black elder include vitamin C. This is an exceptionally labile compound, susceptible to temperature distributions and strong antioxidants. It was observed that in the final syrups obtained by applying extraction at a temperature of 30 °C to flowerheads of black elder treated with ozone, the contents of this important component increase in comparison to the control sample (Figure 2). The findings show the dependence between the contents of vitamin C and the dose of ozone (concentration and duration of the treatment). It was shown that the most beneficial effect is obtained in syrups produced from flowerheads treated with ozone for 5 min at a rate of 100 ppm; the contents of vitamin C in the product of this process are 56.5% higher than in the control syrup. A high dose of ozone was also absorbed by flowerheads treated for 30 min at a rate of 10 ppm, which led to an increase in vitamin C content to the level of 26.56 mg dm^−3^ of the syrup. Similar relationships were observed in the case of syrups produced from treated raw material with the use of sugar syrup at a temperature of 60 °C. In this case, however, the highest content of vitamin C was identified in syrup made from flowers of black elder treated with ozone for 15 min, irrespective of the concentration applied. Notably, the highest content of vitamin C, amounting to 46.17 mg dm^−3^, was identified in the sample of syrup made from elderflowers treated with ozone for 15 min at a rate of 10 ppm by adding sugar syrup with a temperature of 60 °C. The relevant value was 58.12% higher, compared to the highest content of vitamin C identified in syrups produced at 30 °C (29.20 mg dm^−3^).

An increase in the content of this component may have been associated with onset of oxidative stress which led to intensified production of antioxidants, including vitamin C. A similar effect was observed when raspberry fruit was stored in an ozone atmosphere. It has been shown that ozone significantly influences the vitamin C content in these fruits [26]. Researchers have also reported increased contents of vitamin C in other materials treated with ozone [37].

### 2.3. Total Contents of Polyphenols in Syrups

**Figure 3:**
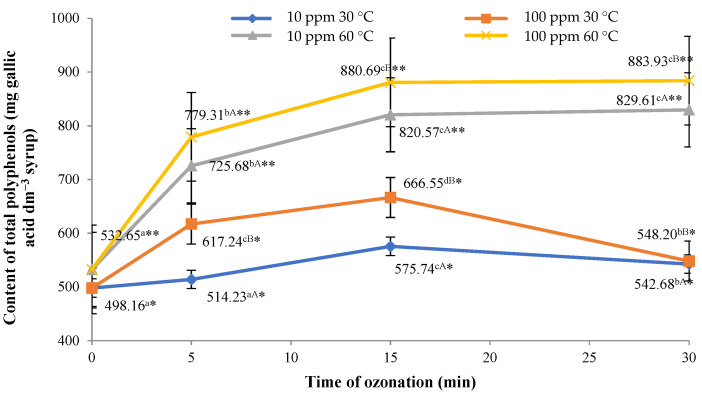
Total contents of polyphenols in syrups from flowers of black elder, relative to the dose of ozone applied and temperature of sugar syrup used. Note: Differences in results between the time of ozonation (for certain ozone concentration) are indicated by different small letters, differences between ozone concentration (for certain ozonation time) are indicated by different capital letters and differences between sugar syrup temperatures are indicated by * or ** (for the same ozone-treatment conditions) at α < 0.05.

Ozone treatment applied to flowerheads of black elder affected the total contents of polyphenols in the syrups, irrespective of the temperature of water used in the process (Figure 3). However, it was found that in the case of syrup produced using sugar syrup at a temperature of 60 °C, a longer duration of the ozone treatment applied to the raw material resulted in a significant increase in the total content of polyphenols in the final product. The highest content of polyphenols was found in syrup made from flowerheads of black elder treated with ozone at a rate of 100 ppm for 30 min and sugar syrup with a temperature of 60 °C. The effect of the higher temperature of water on the content of polyphenols in aquatic extracts of chamomile (*Matricaria chamomilla* L.) was reported by Harbourne et al. [38]. Based on the tests carried out, these authors concluded that extraction of all the components occurs in accordance with pseudo-first-order kinetics. The rate constant (k) grew with an increase in temperature from 57 to 100 °C in line with the van’t Hoff rule; hence, aqueous chamomile extracts had the maximum total concentration of phenol following extraction at 90 °C. Although the temperature range of 30–60 °C used is different from that given by Harbourne et al. [38], the results obtained are similar, i.e., increasing the temperature caused a significant increase in the concentration of the isolated components.

The increase in the contents of polyphenols, identified in all the samples of syrup made from flowerheads of black elder, irrespective of the temperature of the sugar syrup added, may have been associated with the phenomenon of elicitation, which leads to activation of defence mechanisms in the raw material in response to oxidative stress induced by a lack of balance between production and neutralisation of reactive oxygen species (ROS), including gaseous ozone (O_3_). A study by Chen et al. [39] showed that ozone treatment applied to strawberries resulted in elevated levels of polyphenols due to increased expression of genes encoding phenylalanine ammonialyase. On the other hand, Piechowiak et al. [40] showed that gaseous ozone applied to raspberries at a concentration of 8–10 mg L^−1^ for 30 min, every 12 h for a period of 72 h, significantly affects the levels of phenolic compounds and glutathione (GSH) in the fruit.

### 2.4. Determination of Polyphenolic Compounds

Biological activity of syrups from flowers of black elder depends on the contents of a few key components, such as vitamin C, volatile substances and polyphenols. As shown by Piechowiak and Balawejder [34], in some plant materials, ozone treatment leads to an increase in the contents of polyphenols. A similar phenomenon was observed in the case of syrups from elderflowers treated with ozone, where the content of this group of compounds was higher than in the control samples. The activity, in particular antioxidant potential, is affected by the total content of polyphenols and their profile.

Ozone is a known abiotic elicitor that may affect the profile of polyphenols (Table 1 and Table 2). It was established that, in favourable conditions, it activates the expression of genes responsible for the biosynthesis of phenylalanine ammonia lyase (PAL; E.C 4.3.1.5). This promotes the activity of the enzyme, which leads to increased biosynthesis of polyphenols. Other enzyme systems were also activated in raspberry fruit: superoxide dismutase, ascorbate peroxidase, which proves the occurrence of oxidative stress. It should be noted, however, that this stress stimulated the plant material to produce low-molecular-weight antioxidants in order to protect against the phytotoxic effects of ozone [32]. Thiyagarajan et al. [41] in their study proposed a pathway for the biosynthesis of quercetin and rutins, which are the main polyphenols identified in flowers. Similar relationships were observed in the case of syrups produced from elderflowers treated with ozone. It was found that the contents of the dominant components, particularly glycosides of quercetin, i.e., rutin, increase with the dose of ozone and temperature of sugar syrup. It was also observed that the contents of aglycones (quercetin) decrease with the dose of ozone and temperature of sugar syrup. These compounds are colouring agents, and because of this, they affect the colour of the final product (Table 3), which is very important from consumers’ viewpoints.

### 2.5. Colour

Polyphenol pigments are the main compounds affecting the colour of syrups. It was shown that rutin, which is a yellow pigment, dominates in the profile of these compounds.

The results of the analyses showed that in ozonated elderberry flowers, the content of dye, mainly in the group of polyphenols, increased as a result of oxidative stress. The effect of increasing the content of polyphenols and changing the colour of raw materials is often observed under the influence of ozone gas. This effect is most often seen in soft fruits. However, other raw materials that carry out gas exchange react in a similar way [42]. Analysis of colour measurements showed a strong association between the content of this compound and the value of parameter b* related to colour; its changes are observed at a shift towards the yellow colour (Table 3). The highest values of parameter b* were identified in the case of syrups made from elderflowers treated with ozone (100 ppm, 15 min) and from sugar syrup with a temperature of 60 °C. High values of this parameter of colour were also identified in the case of syrup made from sugar syrup with a temperature of 30 °C and from flowers treated with ozone for 5 or 15 min at a rate of 100 ppm. Syrups of this kind have a characteristic strong yellow colour. It was shown that ozone treatment applied to raw material used for this type of product may favourably affect their quality parameters, including colour. The findings also show changes in colour parameter a* whose values ranged from −2.76 to 5.67. However, the narrow range of the changes in this parameter corresponded to only mild effects reflected by the colour of the syrups. The luminosity of colour in the syrups, measured with parameter L*, was in the narrow range of 58.45–77.48, which was reflected by visual similarity of the syrups.

### 2.6. Profile of Volatile Compounds

Syrup made from flowers of black elder is intended for consumption. The flavour of such products is largely determined by volatile substances (Table 4). These compounds are mainly contained in the raw material as components of its essential oil. Because of the high content of sugar in the produced syrups, components of the volatile fraction were identified using HS–SPME method. It was found that the headspace phase is rich in volatile derivatives of terpenes, including mainly aerobic derivatives. There were significant quantitative changes in the contents of volatile substances investigated. The findings showed a relationship between the dose of ozone (concentration and duration) and the quantity of cyclic derivatives of linalool ((*Z*)-linalool oxide and linalool oxide). The first stage in the formation of these derivatives involves epoxidation and then cyclisation into a furanoid form ((*Z*)-linalool oxide) and pyranoid form (linalool oxide). Ozone is a known epoxidiser [43], which as a consequence leads to further cyclisation [44]. The cyclic derivatives produced in the process present good sensory properties favourably affecting the flavour profile of the syrups investigated in the study. Notably, a similar process has been observed in natural processes of linalool biotransformation with the use of fungus *Aspergillus niger* and *Corynespora cassiicola* [44]. Ozone treatment also significantly affected the contents of isomenthyl acetate; its content decreases with the applied dose of ozone. These relationships were observed in the case of syrups produced using sugar syrups at both temperatures, i.e., 30 °C and 60 °C.

## 3. Materials and Methods

### 3.1. Plant Materials

Plant material used in the experiment consisted of fresh flowers of black elder plants (*Sambuci flos*) growing in the wild. The material was collected from shrubs growing in a natural stand in Poland (50°03′37.0″ N 22°00′52.4″ E). *Sambuci flos* with uniform colour was collected during the first quarter of June 2020. The mean weight of the fresh flowerheads was 63.12 ± 14.06 g. Content of dry weight in the raw material was 23.76 ± 0.63%.

### 3.2. The Ozone Treatment of the Plant Material

Samples of flowers of a black elder were placed into the ozonation chamber. This device was used in combination with the Ozone Solution TS 30 ozone generator, and the ozone concentration was measured by the 106 M UV-Ozone Solution detector (Ozone Solution, Hull, IA, USA), with a measuring range of 0–1000 ppm. Flowers of black elder were exposed to gaseous ozone at the concentration of 10 ppm and 100 ppm for 5, 15 and 30 min utilising a 4 m^3^ h^−1^ flow rate of ozone. The ozonation process was carried out at constant temperature of 20 °C, in three replications, and was later produced of syrups from flowers of black elder.

### 3.3. Production of Syrups from Flowers of Black Elder

Samples comprising 300 g of flower heads from black elder, previously subjected to ozone treatment, were placed in a vessel made from smoked glass along with 500 mL of sugar syrup, which had been produced by dissolving 1.5 kg of sucrose in 1 dm^3^ of hot water. The final syrup, obtained using sugar syrup at a temperature of 30 °C or 60 °C, was left to be extracted for two weeks at a constant temperature of 25 °C and humidity of 80%. After that time, the solid parts were removed by filtration as a result of which viscous, straw-coloured liquid with characteristic scent was obtained. Subsequently, the final syrups were subjected to chemical analyses.

### 3.4. Determination of Antioxidant Activity

Elderberry flower syrup (0.5 mL) was diluted twice with methanol, vortexed and centrifuged at 7500× *g* for 15 min. The solution was subjected to further antioxidant activity and polyphenols analysis.

The antioxidant activity of elderberry flower syrup was assayed with the CUPRAC method presented by Piechowiak et al. [40] with minor modifications. Briefly, 10 μL of elderberry extract, 40 μL of 10 mM CuCl_2_, 50 μL of 7.5 mM neocuproine and 50 μL of 1 M NH_4_Ac were added to a well plate. After 30 min of incubation in darkness, the absorbance at λ = 450 nm was measured using the EPOCH microplate reader (Biotek, Puławy, Poland). The results obtained were expressed as quercetin equivalent (mg) per 1 dm^3^ of syrup.

### 3.5. Determination of Ascorbic Acid Content

Vitamin C estimation was determined by the Folin–Ciocalteu reagent method by Porter [45]. Elderberry flower syrup (0.5 mL) was diluted with 0.5 mL of 1% trichloroacetic acid, vortexed for 30 s and centrifuged at 7500 g for 15 min. Next, to 50 μL of the supernatant obtained, 25 μL of Folin–Ciocalteu reagent and 75 μL of 10% TCA were added. After incubation (30 min), the absorbance at λ = 760 nm was measured using a microplate reader. The results obtained were expressed as mg of vitamin C per 1 dm^3^ of syrup.

### 3.6. Total Phenolic Content Assay

The level of total phenolic content was analysed according to the method presented by Piechowiak et al. [30]. Briefly, 20 μL of the sample was mixed with 80 μL of distilled water, 20 μL of Folin–Ciocalteu reagent and 80 μL of 20% Na_2_CO_3_. After incubation in darkness (30 min), the absorbance at λ = 700 nm was measured. The results were expressed as gallic acid equivalent (mg) per 1 L of syrup.

### 3.7. Polyphenolic Compounds Analysis

#### 3.7.1. Sample Preparation

Ten millilitres of elderberry flowers syrup was diluted with 10 mL of distilled water. The obtained solution was passed through a C18 Sep-Pack cartridge (Waters Associates, Milford, MA, USA) preconditioned with water. The cartridge was washed first with water to remove sugars and polar compounds, and then polyphenols compounds were eluted with methanol. The eluates were collected and evaporated to dryness at 40 °C using a rotary evaporator (R-215 Rotavapor System, Büchi, Switzerland). The dry residue was dissolved in 1 mL of acetonitrile (50% *v/v*) and then diluted in water in a 1:5 ratio. The solution has been transferred to a chromatographic vial and stored at 4 °C until chromatographic analysis.

#### 3.7.2. Determination of Polyphenols Profile

Determination of polyphenolic compounds was carried out using the ultra-performance liquid chromatography (UPLC) Waters ACQUITY system (Waters, Milford, MA, USA). The UPLC system was equipped with a binary pump manager, column manager, sample manager, photodiode array (PDA) detector and tandem quadrupole mass spectrometer (TQD) with an electrospray ionisation (ESI) source. Separation of polyphenols was performed using a 1.7 µm, 100 mm × 2.1 mm UPLC BEH RP C18 column (Waters, USA). For the anthocyanin investigation, the mobile phase consisted of 2% formic acid in water, *v/v* (solvent A) and 2% formic acid in 40% acetonitrile, *v/v* (solvent B). However, in the case of other polyphenolic compounds, water (solvent A) and 40% acetonitrile, *v/v* (solvent B) were used. The flow rate was kept constant at 0.35 mL/min for a total run time of 8 min. The system was run with the following gradient program: from 0 min 5% B, from 0 to 8 min linear to 100% B and from 8 to 9.5 min for washing and back to initial conditions. The injection volume of the samples was 5 µL, and the column was supported at 50 °C. The following TQD parameters were used: cone voltage of 30 V, capillary voltage of 3500 V, source and desolvation temperature 120 °C and 350 °C, respectively, and desolvation gas flow rate of 800 L/h. Characterisation of the individual polyphenolic compounds was performed on the basis of the retention time, mass-to-charge ratio, fragment ions and comparison of data obtained with commercial standards and literature findings. Obtained data were processed in Waters MassLynx v.4.1 software (Waters, USA).

### 3.8. Head Space–Solid Phase Microextraction HS–SPME of Prepared Syrup

In order to determine the profile of volatile substances contained in the syrups from flowers of black elder, a 5 mL sample was placed in a bottle with a rubber septum and thermally stabilised at 40 °C for 15 min, in order to achieve maximum vapour pressure of volatile substances. Subsequently, the analytes were separated using a 100 μm polydimethylsiloxane (PDMS) fibre manufactured by Supelco Ltd. (Bellefonte, PA, USA) through exposition of the fibre for 30 min.

### 3.9. Chromatographic Analysis

Composition of the desorbed compounds was examined using gas chromatograph Varian 450 GC with the mass detector (GC-MS) Varian 240 MS, according to the method proposed by Matłok et al. [46].

### 3.10. Colour Change

The prepared syrup was measured in a Commission Internationale de l’Eclairage (CIE) L*a*b* system reflected in light using a Colour Ques spectrophotometer (HunterLab, Reston, VA USA), according to the method proposed by Matłok et al. [47].

### 3.11. Statistical Analysis

Multidimensional analysis of variance (ANOVA) of results was conducted at the significance level α = 0.05 utilising STATISTICA 13.1 software (TIBCO Software Inc., Hillview Avenue, Palo Alto, CA, USA). The mean values calculated from the three independent replications were analysed statistically by comparing the results between the variants of the experiment.

## 4. Conclusions

Syrups produced from ozonated flowers of black elder are a good source of bioactive compounds, as they contain an increased amount of compounds from various groups, mainly polyphenols, vitamin C and volatile substances, which determine the aroma of the products. The contents of these compounds in the final product may be fortified by applying ozone treatment to the raw plant material and by using sugar syrup at various temperatures in the production process. The findings show a significant increase in the contents of the bioactive compounds in the syrups produced from raw material treated with ozone for 15 min at a rate of 10 ppm. In the case of syrups produced at a temperature of 30 °C, there was also a beneficial effect of ozone treatment applied to raw plant material; however, the contents of bioactive compounds in the final syrups were lower than in the syrups produced at a temperature of 60 °C. The colour of the syrups depended on the content of rutin and was most intensive in the case of syrups made from elderflowers treated with ozone (100 ppm, 15 min). It should be noted that, regardless of the extraction temperature, increased levels of bioactive compounds from ozonated elderberry, flowers were isolated. Analysis of volatile substances showed that the contents of linalool, cyclising into (*Z*)-linalool oxide and linalool oxide, decrease with the dose of ozone (duration and concentration).

## Figures and Tables

**Table 1:** Content (%) of individual phenolic compounds identified by UPLC–PDA–MS/MS in a syrup prepared with sugar syrup at a temperature of 30 °C.

Compound		Rt	λ_max_	(M − H) *m/z*		Content in Syrup (%)						
		min	nm	MS	MS/MS	0 ppm	10 ppm			100 ppm		
						0 min	5 min	15 min	30 min	5 min	15 min	30 min
1	Chlorogenic acid	2.86	298 sh/328	353	191/179	0.70 ± 0.03 ^a^	1.08 ± 0.16 ^a^	3.45 ± 0.30 ^c^	3.60 ± 0.13 ^c^	1.40 ± 0.03 ^a^	2.16 ± 0.71 ^b^	2.77 ± 0.41 ^b^
2	Unspecified hydroxybenzoic derivative	3.09	264	393	163	0.68 ± 0.25 ^b^	0.74 ± 0.18 ^b^	1.07 ± 0.21 ^c^	1.67 ± 0.05 ^c^	1.06 ± 0.09 ^c^	1.30 ± 0.12 ^c^	0.14 ± 0.04 ^a^
3	Naringenin 5,7-*O*-di-glucoside	3.13	268/324	595	271	0.16 ± 0.01 ^a^	0.18 ± 0.02 ^a^	0.54 ± 0.53 ^b^	0.89 ± 0.06 ^c^	0.49 ± 0.03 ^b^	0.81 ± 0.01 ^c^	0.39 ± 0.04 ^b^
4	Naringenin 7-*O*-rutinoside-5-*O*-pentoside	3.24	271/317	711	403/271	0.27 ± 0.03 ^a^	0.25 ± 0.01 ^a^	0.73 ± 0.68 ^b^	0.69 ± 0.24 ^b^	0.99 ± 0.36 ^b^	1.01 ± 0.23 ^b^	0.70 ± 0.17 ^b^
5	Kaempferol 3-*O*-di-glucoside	3.30	262/317	609	285	0.17 ± 0.02 ^a^	0.25 ± 0.02 ^a^	0.45 ± 0.09 ^b^	0.57 ± 0.00 ^b^	0.43 ± 0.26 ^b^	0.51 ± 0.01 ^b^	0.13 ± 0.01 ^a^
6	Quercetin 3-*O*-rutinoside-7-*O*-glucoside	3.57	255/347	771	609/301	0.44 ± 0.16 ^b^	0.27 ± 0.08 ^a^	0.24 ± 0.15 ^a^	0.56 ± 0.00 ^b^	1.10 ± 0.57 ^d^	0.71 ± 0.06 ^c^	0.40 ± 0.11 ^b^
7	Quercetin 3-*O*-di-glucoside	3.77	255/352	625	301	4.87 ± 0.86 ^a^	4.56 ± 0.71 ^a^	4.08 ± 0.93 ^a^	4.98 ± 0.46 ^a^	6.22 ± 1.54 ^b^	6.91 ± 1.45 ^b^	4.96 ± 1.27 ^a^
8	Quercetin 3-*O*-rutinoside-7-*O*-pentoside	3.88	255/355	741	609/301	0.15 ± 0.02 ^b^	0.08 ± 0.01 ^a^	0.06 ± 0.01 ^a^	0.41 ± 0.11 ^c^	0.39 ± 0.20 ^c^	0.19 ± 0.05 ^b^	0.06 ± 0.01 ^a^
9	Quercetin 3-*O*-rutinoside-7-*O*-rhamnoside	3.99	255/345	755	609/301	1.15 ± 0.04 ^a^	1.22 ± 0.03 ^a^	1.33 ± 0.31 ^a^	1.07 ± 0.18 ^a^	1.20 ± 0.20 ^a^	1.08 ± 0.14 ^a^	1.15 ± 0.02 ^a^
10	Quercetin 3-*O*-glucoside-pentoside	4.05	255/345	595	301	2.64 ± 0.00 ^b^	2.40 ± 0.07 ^b^	2.49 ± 1.10 ^b^	2.18 ± 0.28 ^b^	2.03 ± 0.13 ^b^	1.45 ± 0.09 ^a^	1.58 ± 0.13 ^a^
11	Kaempferol 3,7-*O*-di-glucoside	4.22	264/339	609	447/285	0.68 ± 0.09 ^b^	0.67 ± 0.01 ^b^	0.67 ± 0.23 ^b^	0.86 ± 0.01 ^c^	0.79 ± 0.27 ^c^	0.59 ± 0.04 ^a^	0.41 ± 0.00 ^a^
12	Quercetin 3-*O*-glucoside-7-*O*-glucuronide	4.30	255/352	639	463/301	4.99 ± 0.78 ^b^	5.69 ± 0.98 ^b^	5.64 ± 2.00 ^b^	5.06 ± 0.41 ^b^	4.01 ± 0.54 ^a^	3.63 ± 1.01 ^a^	3.32 ± 0.72 ^a^
13	Quercetin 3-*O*-rutinoside (Rutin)	4.38	255/352	609	301	22.90 ± 0.53 ^a^	19.57 ± 1.3 ^a^	20.94 ± 0.37 ^a^	23.30 ± 0.43 ^a^	32.73 ± 1.20 ^b^	27.82 ± 2.01 ^b^	35.63 ± 1.42 ^b^
14	Quercetin 3-*O*-glucoside	4.61	255/355	463	301	8.21 ± 1.35 ^b^	6.93 ± 0.78 ^a^	6.72 ± 0.01 ^a^	7.17 ± 0.10 ^a^	9.41 ± 0.29 ^b^	11.31 ± 0.79 ^c^	9.04 ± 2.23 ^b^
15	Quercetin 3-*O*-(6″-acetyl)-glucoside	4.96	255/352	505	463/301	8.45 ± 0.20 ^d^	9.66 ± 1.46 ^d^	8.60 ± 0.06 ^d^	8.68 ± 0.31 ^d^	4.89 ± 0.23 ^b^	6.96 ± 0.36 ^c^	3.20 ± 0.52 a
16	3,4-dicaffeoylquinic acid	5.14	288 sh/328	515	353/191	18.54 ± 0.46 ^b^	19.75 ± 0.78 ^b^	21.29 ± 2.05 ^b^	16.12 ± 0.96 ^a^	17.48 ± 0.54 ^a^	15.55 ± 0.48 ^a^	18.98 ± 1.87 ^b^
17	Quercetin 3-*O*-glucuronide	5.32	255/348	477	301	13.45 ± 1.27 ^b^	15.22 ± 1.03 ^b^	10.11 ± 1.77 ^a^	12.36 ± 0.73 ^b^	8.10 ± 2.69 ^a^	9.90 ± 0.94 ^a^	9.55 ± 0.54 ^a^
18	1.5-di-caffeoyl-quinic acid	5.48	288 sh/326	515	353/179	1.05 ± 0.18 ^b^	1.21 ± 0.27 ^b^	1.31 ± 0.11 ^b^	0.78 ± 0.02 ^a^	0.74 ± 0.35 ^a^	2.19 ± 0.01 ^c^	2.05 ± 0.06 ^c^
19	Kaempferol 3-*O*-rhamnoside-7-*O*-pentoside	5.66	264/345	563	431/285	3.82 ± 0.06 ^c^	5.30 ± 1.01 ^d^	5.38 ± 0.82 d	4.11 ± 0.05 d	2.15 ± 0.15 ^a^	2.53 ± 0.19 ^b^	1.86 ± 0.10 ^a^
20	Unspecified caffeoyl-quinic derivative	6.55	288 sh/322	538	341/191	1.16 ± 0.24 ^a^	1.42 ± 0.41 ^a^	1.54 ± 0.01 ^a^	1.88 ± 0.07 ^b^	1.23 ± 0.21 ^a^	1.41 ± 0.09 ^a^	1.56 ± 0.70 ^a^
21	Quercetin	6.62	255/355	301	-	3.47 ± 0.21 ^d^	2.09 ± 0.11 ^c^	1.96 ± 0.24 ^b^	1.91 ± 0.11 ^b^	1.79 ± 0.04 ^a^	1.47 ± 0.03 ^a^	1.56 ± 0.27 ^a^
22	Naringenin	7.53	271/317	271	-	0.85 ± 0.04 ^b^	1.31 ± 0.01 ^c^	1.18 ± 0.02 ^c^	0.87 ± 0.07 ^b^	1.11 ± 0.21 ^c^	0.36 ± 0.00 ^a^	0.35 ± 0.00 ^a^
23	Quercetin 7-methyl ether	7.98	255/355	315	-	1.22 ± 0.07 ^b^	0.16 ± 0.00 ^a^	0.20 ± 0.11 ^a^	0.29 ± 0.17 ^a^	0.25 ± 0.04 ^a^	0.17 ± 0.02 ^a^	0.21 ± 0.18 ^a^

RT—retention time; sh = shoulder peak; ±SD and *n* = 3; FD. Note. Differences in results between the dose of ozone are indicated by different small letters, difference at significant level α < 0.05.

**Table 2:** Content (%) of individual phenolic compounds identified by UPLC–PDA–MS/MS in a syrup prepared with sugar syrup at a temperature of 60 °C.

Compound		Rt	λ_max_	(M − H) *m/z*		Content in Syrup (%)						
		min	nm	MS	MS/MS	0 ppm	10 ppm			100 ppm		
						0 min	5 min	15 min	30 min	5 min	15 min	30 min
1	Chlorogenic acid	2.86	298 sh/328	353	191/179	5.43 ± 0.79 ^b^	4.35 ± 0.63 ^b^	3.77 ± 0.20 ^a^	3.70 ± 0.61 ^a^	5.58 ± 0.50 ^b^	3.14 ± 0.42 ^a^	2.97 ± 0.92 ^a^
2	Unspecified hydroxybenzoic derivative	3.09	264	393	163	0.99 ± 0.30 ^b^	0.69 ± 0.04 ^a^	0.73 ± 0.00 ^a^	0.80 ± 0.11 ^b^	0.87 ± 0.04 ^b^	0.65 ± 0.00 ^a^	0.80 ± 0.17 ^b^
3	Naringenin 5,7-*O*-di-glucoside	3.13	268/324	595	271	0.83 ± 0.15 ^b^	0.66 ± 0.10 ^a^	0.57 ± 0.06 ^a^	0.63 ± 0.07 ^a^	0.83 ± 0.30 ^b^	0.58 ± 0.05 ^a^	0.62 ± 0.3^1 a^
4	Naringenin 7-*O*-rutinoside-5-*O*-pentoside	3.24	271/317	711	403/271	0.38 ± 0.15 ^a^	0.61 ± 0.01 ^b^	0.78 ± 0.04 ^b^	0.70 ± 0.09 ^b^	1.31 ± 0.06 ^c^	0.80 ± 0.17 ^b^	0.76 ± 0.05 ^b^
5	Kaempferol 3-*O*-di-glucoside	3.30	262/317	609	285	0.14 ± 0.02 ^a^	0.26 ± 0.10 ^b^	0.42 ± 0.12 ^d^	0.39 ± 0.06 ^c^	0.41 ± 0.14 ^d^	0.20 ± 0.07 ^b^	0.33 ± 0.01 ^c^
6	Quercetin 3-*O*-rutinoside-7-*O*-glucoside	3.57	255/347	771	609/301	0.28 ± 0.04 ^a^	0.25 ± 0.05 ^a^	0.40 ± 0.04 ^b^	0.73 ± 0.45 ^c^	1.37 ± 0.57 ^d^	0.85 ± 0.14 ^c^	1.15 ± 0.05 ^d^
7	Quercetin 3-*O*-di-glucoside	3.77	255/352	625	301	3.21 ± 0.17 ^a^	3.98 ± 0.24 ^a^	4.45 ± 0.32 ^b^	4.95 ± 0.17 ^b^	4.74 ± 0.06 ^b^	4.78 ± 1.91 ^b^	4.52 ± 0.90 ^b^
8	Quercetin 3-*O*-rutinoside-7-*O*-pentoside	3.88	255/355	741	609/301	0.10 ± 0.01 ^a^	0.08 ± 0.00 ^a^	0.07 ± 0.03 ^a^	0.25 ± 0.20 ^b^	0.26 ± 0.24 ^b^	0.08 ± 0.03 ^a^	0.30 ± 0.12 ^b^
9	Quercetin 3-*O*-rutinoside-7-*O*-rhamnoside	3.99	255/345	755	609/301	2.02 ± 0.27 ^b^	1.60 ± 0.15 ^a^	1.72 ± 0.15 a	1.89 ± 0.27 a	2.13 ± 0.90 b	1.49 ± 0.13 a	1.86 ± 0.38 a
10	Quercetin 3-*O*-glucoside-pentoside	4.05	255/345	595	301	2.11 ± 0.12 ^b^	1.85 ± 0.07 ^a^	1.60 ± 0.19 ^a^	2.05 ± 0.06 b	1.74 ± 1.10 a	1.10 ± 0.49 a	2.12 ± 0.48 b
11	Kaempferol 3,7-*O*-di-glucoside	4.22	264/339	609	447/285	0.94 ± 0.00 ^b^	0.65 ± 0.05 ^a^	0.78 ± 0.02 ^b^	0.75 ± 0.03 b	0.53 ± 0.26 a	0.47 ± 0.01 a	0.88 ± 0.35 b
12	Quercetin 3-*O*-glucoside-7-*O*-glucuronide	4.30	255/352	639	463/301	2.89 ± 0.14 ^a^	4.14 ± 0.13 ^b^	4.10 ± 0.25 ^b^	4.42 ± 0.09 b	2.72 ± 0.35 a	2.99 ± 0.22 a	2.81 ± 0.11 a
13	Quercetin 3-*O*-rutinoside (Rutin)	4.38	255/352	609	301	25.41 ± 1.58 ^a^	26.31 ± 0.06 ^a^	24.33 ± 0.13 ^a^	25.13 ± 0.77 ^a^	33.89 ± 8.25 ^b^	39.95 ± 4.28 ^b^	35.76 ± 3.03 ^b^
14	Quercetin 3-*O*-glucoside	4.61	255/355	463	301	11.79 ± 0.13 ^b^	9.20 ± 0.58 ^a^	10.83 ± 0.22 ^b^	8.85 ± 0.01 ^a^	10.17 ± 1.99 ^b^	8.82 ± 3.82 ^a^	8.76 ± 0.67 ^a^
15	Quercetin 3-*O*-(6″-acetyl)-glucoside	4.96	255/352	505	463/301	7.83 ± 0.57 ^c^	7.93 ± 0.28 ^c^	8.83 ± 0.33 ^c^	9.11 ± 0.03 ^c^	3.49 ± 1.00 ^a^	3.74 ± 0.29 ^a^	5.24 ± 2.33 ^b^
16	3,4-dicaffeoylquinic acid	5.14	288 sh/328	515	353/191	15.58 ± 0.13 ^a^	18.19 ± 0.61 ^a^	16.34 ± 0.32 ^a^	16.51 ± 0.18 ^a^	16.36 ± 3.25 ^a^	18.39 ± 2.02 ^a^	17.15 ± 0.13 ^a^
17	Quercetin 3-*O*-glucuronide	5.32	255/348	477	301	12.90 ± 0.66 ^b^	11.41 ± 0.29 ^b^	12.35 ± 0.67 ^b^	10.94 ± 0.94 ^b^	8.09 ± 3.33 ^a^	7.00 ± 0.82 ^a^	7.46 ± 1.40 ^a^
18	1.5-di-caffeoyl-quinic acid	5.48	288 sh/326	515	353/179	1.04 ± 0.04 ^a^	1.12 ± 0.03 ^a^	1.09 ± 0.09 ^a^	1.04 ± 0.24 ^a^	1.00 ± 0.01 ^a^	1.13 ± 0.09 ^a^	2.02 ± 0.52 ^b^
19	Kaempferol 3-*O*-rhamnoside-7-*O*-pentoside	5.66	264/345	563	431/285	2.95 ± 0.12 ^b^	3.79 ± 0.26 ^b^	3.82 ± 0.14 ^b^	3.69 ± 0.40 ^b^	1.68 ± 0.56 ^a^	1.50 ± 0.13 ^a^	1.71 ± 0.62 ^a^
20	Unspecified caffeoyl-quinic derivative	6.55	288 sh/322	538	341/191	1.07 ± 0.17 ^a^	1.11 ± 0.10 ^a^	1.43 ± 0.00 ^b^	1.61 ± 0.51 ^b^	0.89 ± 0.09 ^a^	0.70 ± 0.09 ^a^	1.01 ± 0.31 ^a^
21	Quercetin	6.62	255/355	301	-	1.24 ± 0.11 ^c^	0.88 ± 0.09 ^b^	0.61 ± 0.11 ^a^	0.82 ± 0.11 ^b^	0.72 ± 0.19 ^a^	0.68 ± 0.10 ^a^	0.84 ± 0.13 ^b^
22	Naringenin	7.53	271/317	271	-	0.60 ± 0.05 ^a^	0.85 ± 0.01 ^b^	0.48 ± 0.39 ^a^	0.92 ± 0.23 ^b^	1.05 ± 0.25 ^b^	0.81 ± 0.26 ^b^	0.84 ± 0.38 ^b^
23	Quercetin 7-methyl ether	7.98	255/355	315	-	0.28 ± 0.04 ^c^	0.07 ± 0.04 ^a^	0.53 ± 0.58 ^d^	0.13 ± 0.04 ^a^	0.16 ± 0.07 ^b^	0.15 ± 0.01 ^b^	0.11 ± 0.00 ^a^

RT—retention time; sh = shoulder peak; ±SD and *n* = 3; FD. Note. Differences in results between the dose of ozone are indicated by different small letters, difference at significant level α < 0.05.

**Table 3:** Colour of syrups from flowers of black elder, relative to the temperature of water and dose of ozone applied to the raw materials.

Temperature of Sugar Syrup	Ozone Concentration (ppm)	Time of Ozonation (min)	L* ± SD	a* ± SD	b* ± SD
30 °C	0	0	69.60 ± 1.01 ^b^*	0.22 ± 0.04 ^c^**	41.87 ± 3.06 ^b^**
	10	5	69.34 ± 0.98 ^bB^*	0.00 ± 0.00 ^bA^**	38.87 ± 2.79 ^bA^*
		15	68.35 ± 1.26 ^bB^*	0.10 ± 0.06 ^bA^**	38.64 ± 1.67 ^bA^*
		30	75.00 ± 1.19 ^cB^*	−1.98 ± 0.16 ^aA^*	31.94 ± 3.27 ^aA^*
	100	5	60.41 ± 0.87 ^aA^*	5.67 ± 0.36 ^dB^**	49.69 ± 2.09 ^cB^*
		15	58.45 ± 0.94 ^aA^*	5.66 ± 0.048 ^dB^*	49.90 ± 1.97 ^cB^*
		30	61.05 ± 2.06 ^aA^*	2.41 ± 0.09 ^bB^*	43.07 ± 3.07 ^bB^*
60 °C	0	0	77.48 ± 2.75 ^b^**	−3.64 ± 0.47 ^a^*	36.61 ± 2.11 a*
	10	5	72.62 ± 1.05 ^bB^**	−2.22 ± 0.07 ^bA^*	36.50 ± 2.97 ^Aa^*
		15	74.18 ± 1.97 ^bB^**	−2.76 ± 0.09 ^bA^*	36.34 ± 1.64 ^aA^*
		30	70.59 ± 0.36 ^bB^*	−1.95 ± 0.04 ^cA^*	36.93 ± 2.07 ^aA^*
	100	5	66.45 ± 1.09 ^aA^**	1.91 ± 0.10 ^bB^*	46.40 ± 2.46 ^bB^*
		15	60.54 ± 0.46 ^aA^*	4.98 ± 1.16 ^cB^*	51.18 ± 2.46 ^bB^*
		30	61.92 ± 0.76 ^aA^*	3.29 ± 0.97 ^cB^**	48.37 ± 1.79 ^bB^*

Note: Differences in results between the time of ozonation (for certain ozone concentration) are indicated by different small letters, and difference between ozone concentration (for certain ozonation time) are indicated by different capital letters and difference between sugar syrup temperatures are indicated by * or ** (for the same ozone-treatment conditions) at α < 0.05.

**Table 4:** Volatile compounds’ composition of syrups produced from black elder.

No.	RT (min)	Peak Share in the Chromatogram (%)														Ordinary Substance Name
		0 ppm 0 min		5 min 10 ppm		15 min 10 ppm		30 min 10 ppm		5 min 100 ppm		15 min 100 ppm		30 min 100 ppm		
		30 °C	60 °C	30 °C	60 °C	30 °C	60 °C	30 °C	60 °C	30 °C	60 °C	30 °C	60 °C	30 °C	60 °C	
1	9.80	trace	trace	trace	trace	trace	trace	trace	trace	trace	trace	trace	trace	trace	trace	benzyl alcohol
2	10.42	19.09 ^bB^	16.56 ^aA^	18.91 ^bA^	16.03 ^aA^	13.85 ^aA^	16.47 ^aB^	26.61 ^cB^	15.49 ^aA^	18.85 ^bA^	21.53 ^bB^	25.37 ^cA^	22.05 ^bA^	26.61 ^cB^	21.77 ^bA^	(*Z*)-linalool oxide
3	10.91	13.28 ^dB^	10.75 ^cA^	2.16 ^aA^	5.82 ^aB^	9.90 ^cA^	8.30 ^bA^	4.35 ^bA^	11.28 ^cB^	9.90 ^cA^	8.56 ^bA^	7.44 ^cA^	9.65 ^bA^	4.34 ^bA^	7.32 ^bB^	linalool
4	11.12	2.36 ^aA^	trace	2.27 ^aA^	trace	2.48 ^aA^	2.26 ^aA^	trace	2.41 ^aA^	2.48 ^aA^	trace	trace	trace	trace	trace	*trans*-rose oxide
5	11.95	1.55 ^aA^	trace	trace	trace	trace	trace	trace	trace	trace	trace	trace	trace	trace	trace	furfural
6	12.14	32.79 ^aA^	36.77 ^aA^	42.19 ^bA^	39.34 ^aA^	46.11 ^bA^	41.12 ^bA^	48.63 ^bB^	43.51 ^bA^	36.11 ^aA^	42.94 ^bB^	46.87 ^bA^	45.19 ^bA^	48.62 ^bA^	45.57 ^bA^	linalool oxide
7	12.86	1.10 ^aA^	3.37 ^bB^	1.86 ^aA^	4.35 ^bB^	1.26 ^aA^	trace	trace	2.37 ^aA^	trace	6.34 ^cA^	5.54 ^bA^	5.03 ^cA^	trace	4.91 ^cA^	coumaran
8	13.04	18.32 ^cA^	16.16 ^bA^	14.77 ^cA^	17.42 ^cB^	15.96 ^cA^	17.25 ^cA^	8.41 ^bA^	15.26 ^bB^	15.96 ^cA^	13.34 ^bA^	4.94 ^aA^	11.06 ^bB^	8.41 ^bA^	7.32 ^aA^	isomenthyl acetate
9	13.26	2.16 ^aA^	2.47 ^bA^	4.25 ^cA^	trace	2.00 ^aA^	2.21 ^bA^	trace	2.18 ^bA^	3.00 ^bB^	1.50 ^aA^	trace	4.91 ^cA^	trace	4.14 ^cA^	(*Z*)-citral
10	14.33	2.40 ^aA^	trace	3.48 ^bA^	4.06 ^bA^	trace	trace	4.08 ^cB^	1.66 ^aA^	3.48 ^bB^	1.36 ^aA^	2.75 ^aA^	trace	4.07 ^cB^	1.55 ^aA^	*p*-vinyl guaiacol
11	14.45	2.76 ^aA^	3.08 ^aA^	2.87 ^aA^	3.75 ^aB^	2.02 ^aA^	3.13 ^aB^	3.70 ^bA^	3.25 ^aA^	3.02 ^bA^	trace	3.75 ^bA^	trace	3.69 ^bA^	trace	4-methoxy-2,3,6-trimethyl-phenol
12	15.05	3.33 ^bB^	1.87 ^aA^	2.73 ^bA^	trace	1.78 ^aA^	1.73 ^aA^	4.22 ^cB^	2.39 ^aA^	3.77 ^cA^	trace	3.33 ^bA^	trace	4.22 ^cA^	4.00 ^bA^	3,4-dimethoxy styrene
TOTAL		99.08 ^bB^	91.03 ^aA^	95.49 ^aB^	90.77 ^aA^	95.36 ^aA^	92.75 ^aA^	100.00 ^bA^	99.80 ^bA^	96.57 ^aA^	95.57 ^bA^	99.99 ^bA^	97.89 ^bA^	99.96 ^bA^	96.58 ^bA^	

Note: Differences in results between the dose of ozone are indicated by different small letters, and differences between temperature of syrup are indicated by different capital letters, difference at significant level *p* < 0.; RT—retention time.

## Data Availability

The data presented in this study are available in this article.

## Sample Availability

Samples of the compounds are not available from the authors.
